# Errata

**Published:** 1889-09

**Authors:** 


Errata.—In our August issue the following errors occur: The remark on p. 314, line 21, and the paragraph on p. 315 (line 12 *et seq.*), are wrongly attributed to Dr. J. B. Bell. The paragraph on p. 315 should be attributed to Dr. W. L. Reed. Page 345, Dr. Cohen's article, line 14 from the bottom, we committed a ludicrous blunder in the interpolation of the unnecessary word "eleven," thus spoiling the sense. Page 346, line 14 from top, should read Arum-triph.$^{cm}$. Page 356, the last paragrah, beginning, "In all cases of diphtheria," is erroneously included in the remarks of Dr. Preston. It should be credited to Dr. C. Carlton Smith.

